# Distinct gene signatures define the epithelial cell features of mucinous appendiceal neoplasms and pseudomyxoma metastases

**DOI:** 10.3389/fgene.2025.1536982

**Published:** 2025-02-13

**Authors:** Carlos Ayala, Anuja Sathe, Xiangqi Bai, Susan M. Grimes, Jeanne Shen, George A. Poultsides, Byrne Lee, Hanlee P. Ji

**Affiliations:** ^1^ Division of Surgical Oncology, Department of Surgery, Stanford University, Stanford, CA, United States; ^2^ Division of Oncology, Department of Medicine, Stanford University School of Medicine, Stanford, CA, United States; ^3^ Department of Pathology, Stanford University School of Medicine, Stanford, CA, United States

**Keywords:** pseudomyxoma, appendiceal adenocarcinoma, single-cell transcriptome, tumor heterogeneity (TH), metastasis

## Abstract

**Introduction:**

Appendiceal mucinous neoplasms (AMN) are rare tumors of the gastrointestinal tract. They metastasize with widespread abdominal dissemination leading to pseudomyxoma peritonei (PMP), a disease with poor prognosis. There are many unknowns about the cellular features of origin, differentiation and progression of AMN and PMP.

**Methods:**

We characterized AMNs, PMPs and matched normal tissues using single-cell RNA-sequencing. We validated our findings with immunohistochemistry, mass spectrometry on malignant ascites from PMP patients and gene expression data from an independent set of PMP tumors.

**Results:**

We identified previously undescribed cellular features and heterogeneity in AMN and PMP tumors. There were gene expression signatures specific to the tumor epithelial cells among AMN and PMP. These signatures included genes indicative of goblet cell differentiation and elevated mucin gene expression. Metastatic PMP cells had a distinct gene expression signature with increased lipid metabolism, inflammatory, JAK-STAT and RAS signaling pathway among others. We observed clonal heterogeneity in a single PMP tumor as well as PMP metastases from the same patient.

**Discussion:**

Our study defined tumor cell gene signatures of AMN and PMP, successfully overcoming challenges of low cellularity and mucinous composition of these tumors. These gene expression signatures provide insights on tumor origin and differentiation, together with the identification of novel treatment targets. The heterogeneity observed within an individual tumor and between different tumors from the same patient, represents a potential source of treatment resistance.

## Introduction

Appendiceal mucinous neoplasms (AMN) are a rare, indolent malignancy that originates in the appendix epithelium. These tumors are notable for their high secretion levels of mucin, which in concert with other components, covers the abdominal cavity’s epithelial surfaces. The excessive secretion is evident in large amounts of gelatin products in the abdomen. AMNs tend to rupture, resulting in intraabdominal dissemination of tumor cells and gelatin. This condition is called pseudomyxoma peritonei (PMP) and is a cause of significant morbidity in patients. Current treatment consists of cytoreductive surgery and heated intraperitoneal chemotherapy (HIPEC) (Choudry and Pai, 2018). However, only 60% of PMP patients are eligible for this treatment (Bartlett et al., 2019). The remainder high-grade PMP patients are treated with systemic chemotherapy albeit with limited benefit for most patients. These poor patient outcomes reflect the extent of metastasis and the specific cellular features of the tumor (Levine et al., 2016). There is a need to identify the specific cellular features and dysregulated biology that distinguish AMN versus PMP tumor cells. Determining these features will be important to identify new and improved therapeutic strategies for patients.

Studying the molecular and cellular features of mucinous tumors of the appendix has been challenging due to their gelatinous nature and low cellularity. As a result, the identification and characterization of the tumor cells responsible for the disease has lagged in comparison to other tumors of the gastrointestinal tract. AMN have histopathology features of goblet cell hyperplasia, but the clinical and biological significance of these cells has not been fully delineated (O'Connell et al., 2002a). Furthermore, elevated mucin production, predominantly MUC2, is detected in AMN and PMP tissues. There is a concept that PMPs may originate from MUC2-expressing goblet or goblet-like cells (O'Connell et al., 2002b). However, PMP tumor epithelial cells do not have the classic histopathologic characteristics of goblet cells precluding their identification as such in pathologic reports (Carr et al., 2017). As a result, little is known about the molecular and cellular features that distinguish AMNs from PMPs tumors.

Genomic studies of PMPs tumors have revealed that the most common mutations are in the *KRAS*, *GNAS* and *TP53* genes (Murage et al., 2023). Other mutations have been described at lower frequencies and lack any biological characterization (Murage et al., 2023). Additional studies have been conducted by Levine et al. (2012), Levine et al. (2016) demonstrating stratification of high and low risk appendiceal clusters of patients based on differential gene expression as compared to colorectal tumors. Additional studies evaluating appendiceal tumors identified a gene signature enriched for oncogenic pathways, which was associated with a worse prognosis (Moaven et al., 2020; Su et al., 2020). A recent study identified that transcriptional signatures of both tumor and fibroblast cells influence PMP prognosis. Importantly, this study identified the need to transcriptionally profile multiple PMP lesions from the same patient to account for their heterogeneity (Isella et al., 2020).

To characterize the gene expression of specific cell populations from both AMN and PMP, we applied single-cell RNA sequencing (scRNA-seq) in a discovery set, followed by independent validation approaches using immunohistochemistry, mass-spectrometry and publicly available data from PMP samples. Our analysis was focused on understanding the origin and differentiation of the tumor epithelial cell populations. This study identified the cell states in tumor epithelium that were associated with the progression of AMN to PMP.

## Methods

### Samples

This study was conducted in compliance with the Helsinki Declaration and approved by the Stanford University School of Medicine Institutional Review Board (IRB-44036). Written informed consent was obtained from all patients. Tissues were collected in plain RPMI on ice immediately after resection and dissected with iris scissors. We prepared single cell suspensions using enzymatic and mechanical dissociation (Miltentyi Biotec, Germany) and generated scRNA-seq libraries (10x Genomics, Pleasanton, CA, United States). Additional details about the experimental and analytical approach are described in the Supplementary Material.

### Batch-corrected integrated analysis and lineage re-clustering

Individual Seurat objects were constructed from each scRNA-seq dataset using Seurat (version 4.0.3) and filtered for low quality cells and computationally identified doublets (Supplementary Material). Objects were merged and normalized using “SCTransform” (Butler et al., 2018; Hafemeister and Satija, 2019). To remove batch effects, we integrated all datasets across experimental batches by using a soft variant of k-means clustering implemented in the Harmony algorithm (Korsunsky et al., 2019) (version 0.1.0). The experimental batch was provided as the grouping variable in the “RunHarmony” function, and this reduction was used in both “RunUMAP” and “FindNeighbors” functions for clustering. The first 20 principal components and a resolution of 1 was used for clustering. We used the Adjusted Rand Index (ARI) to compare similarity between cluster labels and experimental batch meta data label for each cell. Vector of these respective class labels was supplied to the “adjustedRandIndex” function in mclust package (version 5.4.7) (Scrucca et al., 2016).

Following identification of cell lineages based on marker gene expression, we performed a secondary clustering analysis of each lineage. We used the same parameters as described above including integration across experimental batches. From the initial clustering run, we identified clusters that belonged to contaminating cell lineages. These cells’ data were combined with their lineage counterparts and then a second clustering run was performed yielding final lineage-specific re-clustering results. After an independent clustering of the epithelial cell data, we identified those tumor samples with low cellularity samples, defined as being less than 10 cells. These samples were eliminated from downstream analysis.

Data from the “RNA” assay was used for all further downstream analysis with other packages, gene level visualization or differential expression analysis. Data was normalized to the logarithmic scale and effects of variation in sequencing depth were regressed out by including “nCount_RNA” as a parameter in the “ScaleData” function. The “DoHeatmap”, “FeaturePlot”, “DimPlot”, “DotPlot”, “VlnPlot” functions were used for visualization.

### Statistics

Differential gene expression was conducted using the “FindAllMarkers” or “FindMarkers” functions in Seurat using Wilcoxon rank sum test. Parameters provided for these functions were genes detected in at least 25% cells. Differential expression threshold of 0.5 log fold change was used in comparison of gene signatures between normal, AMN and PMP. Ribosomal genes were excluded in this analysis. Significant genes were determined with p-value <0.05 following Bonferroni correction. Differential pathways were determined using ANOVA with *post hoc* Tukey Honestly significant difference (HSD). Adjusted p-value <0.05 was the threshold to be significant.

## Results

### Study overview

**FIGURE 1:**
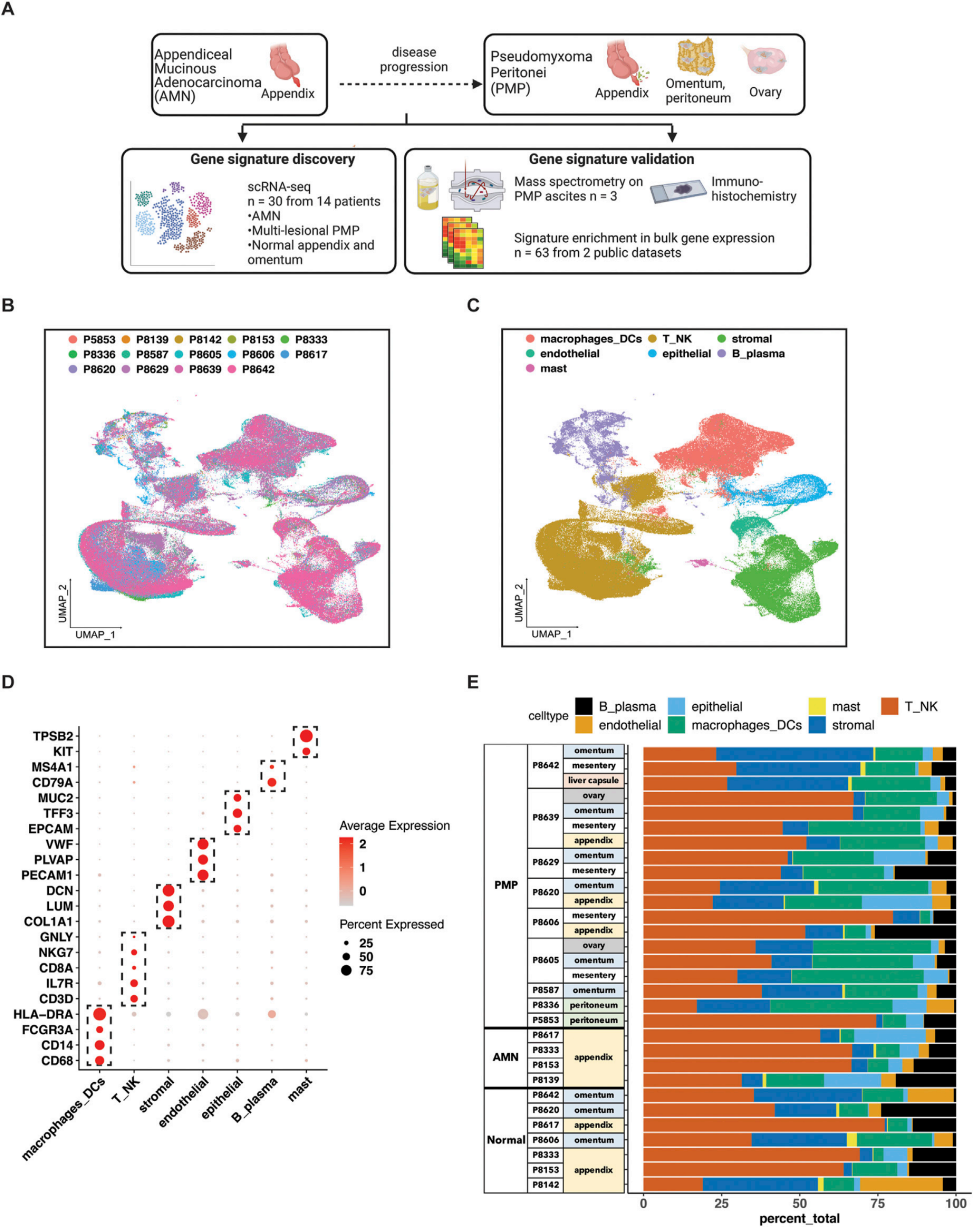
scRNA-seq analysis of AMN and PMP samples. **(A)** Schematic representation of study design. **(B, C)** UMAP representation of dimensionally reduced data following batch correction and graph-based clustering of all datasets annotated by **(B)** samples and **(C)** cell lineages. **(D)** Dot plot depicting average expression levels of specific lineage-based marker genes together with the percentage of cells expressing the marker. **(E)** Proportion of cell lineages detected from each sample annotated by normal, AMN and PMP designation.

Our study focused exclusively on low and high grade appendiceal mucinous neoplasms. This study included AMNs without evidence of visceral perforation and PMPs of appendiceal origin after perforation (Supplementary Table S1). Following surgical resection, all specimens were reviewed by a pathologist at our institution to confirm the site of origin and establish a pathological diagnosis. The AMNs were low-grade appendiceal mucinous neoplasms (LAMN) restricted to the appendix without rupture or peritoneal dissemination. The PMPs, in accordance with PSOGI classification (Carr et al., 2016), were low-grade mucinous carcinoma peritonei (LG-PMP, LAMN) or high-grade mucinous carcinoma peritonei (HG-PMP, mucinous adenocarcinoma or MACA) of the appendix with peritoneal dissemination (Supplementary Figure S1A). Goblet cell adenocarcinoma and tumor with signet ring cell histology were not included in our study. We used multiple approaches to characterize the cellular features of this disease (Figure 1A). We used scRNA-seq to analyze discovery cohort of 14 patients containing 30 samples from AMNs, PMPs and a subset of matched normal appendix or omental tissue. A subset of the PMP samples represented multiple metastatic sites from the same patient. Tumor sites included the omentum, small bowel mesentery, liver capsule, and ovaries.

We validated our findings for specific cell populations using immunohistochemistry (IHC) testing of tumor samples. We also conducted mass spectrometry on ascites samples from three PMP patients to determine if critical gene expression markers could be detected as secreted proteins. Finally, we analyzed an independent cohort of 63 PMP tumors and validated the gene signatures identified in our discovery cohort.

### Single cell RNA-seq profiling of AMN and PMP tumors

Samples included normal appendix, normal omentum, AMNs or PMPs. The scRNA-seq analysis from all samples provided a total of 299,718 cells (Supplementary Table S2). We applied the Harmony algorithm to correct for batch variance in the data (Korsunsky et al., 2019). We observed that each cluster had cells from different patient tumors, indicating adequate removal of noise from experimental batch effects (Figure 1B). We verified this computationally by calculating a similarity metric called the Adjusted Rand Index (ARI) (Hubert and Arabie, 1985). Comparison of cluster assignments with experimental batch had an ARI of 0.05. The low value confirmed that cluster assignments were the result of intrinsic tumor cell properties and not unduly influenced by batch effects.

For each cluster, we assigned cell types based on the expression of established marker genes (Figures 1C, D). This identified epithelial (*EPCAM*, *TFF3*, *MUC2*), stromal fibroblasts (*DCN*, *COL1A1*, *LUM*), endothelial (*VWF*, *PLVAP*, *PECAM1*), T (*CD3D*, *IL7R*, *CD8A*), NK (*NKG7*, *GNLY*), B or plasma (*MS4A1*, CD79A), mast (*TPSAB1*, *KIT*) and macrophage or dendritic cells (*CD68*, *CD14*, *FCGR3A*, *HLA-DRA*). Major lineage types were identified in varying proportions across all samples (Figure 1E). Next, we performed a secondary clustering analysis with batch-correction for each cell lineage, enabling the detailed characterization of its gene expression phenotypes.

### Identifying the epithelial cell subtypes in AMNs and PMPs

**FIGURE 2:**
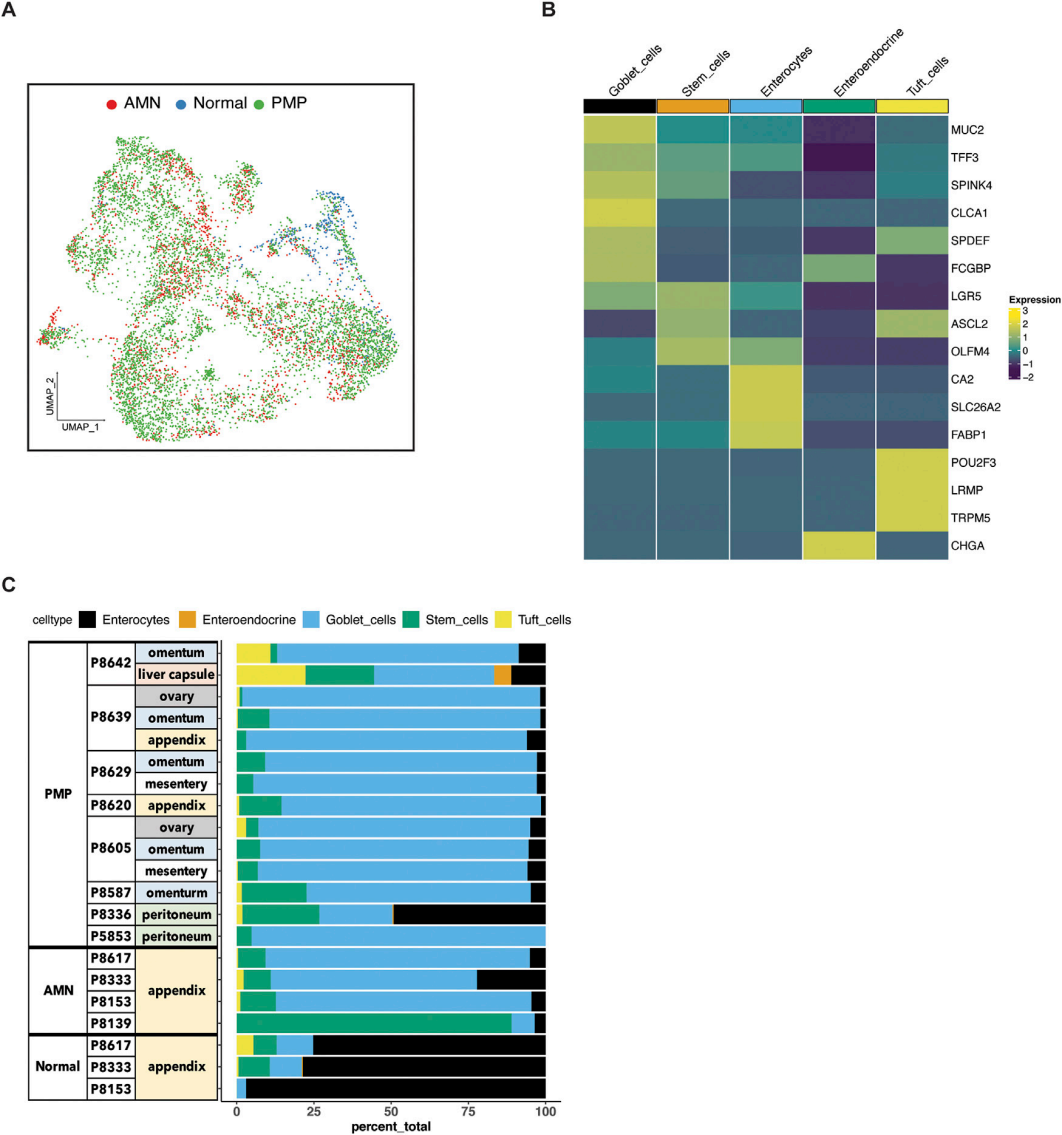
Epithelial cell subtypes in AMN and PMP. **(A)** UMAP representation of dimensionally reduced data following batch correction and graph-based clustering of epithelial cells from all datasets annotated by condition. **(B)** Heatmap depicting average expression of lineage marker genes following reference-based assignment of tumor and normal epithelial cells. **(C)** Proportion of epithelial cell lineages detected from each sample annotated by normal, AMN and PMP designation.

We determined the characteristics of the different epithelial subtypes present in these tumors. We conducted an independent clustering analysis of the epithelial cells from the AMNs, PMPs and normal appendix including samples containing at least 10 cells (Figure 2A; Supplementary Table S3). This threshold of epithelial cell numbers was required to accurately evaluate differential gene expression. Based on this quality control procedure we had 21 tumor samples from 12 patients. Next, we compared the gene expression of each individual cell to a reference single cell atlas of mucosal cell lineages of the human large intestine (Elmentaite et al., 2021). This step relied on the SingleR method which identifies cell types in a query dataset using specific references of gene expression (Supplementary Material) (Aran et al., 2019).

Next, we confirmed the assignment of the cell type by examining the expression of established lineage marker genes. For all tumors, we identified enterocytes (*CA2*, *SLC26A2*, *FABP1*), stem cells (*LGR5*, *ASCL2*, *OLFM4*) and goblet cells (*MUC2*, *TFF3*, *SPINK4*, *CLCA1*, *SPDEF*, *FCGBP*) (Figure 2B). Also, we uncovered rare cell types of the gastrointestinal system which included chemosensory tuft (*POU2F3*, *LRMP*, *TRPM5*) and neuroendocrine cells (*CHGA*).

To compare the composition of normal tissue versus AMN or PMP, we conducted a statistical analysis for differential cell type abundance using a generalized linear model (Supplementary Material) (Haber et al., 2017; Ramachandran et al., 2019). Goblet cells were the major epithelial cells present in both AMN and PMP tumors (Figure 2C). Compared to normal appendiceal tissue, goblet cells were significantly more abundant in both PMPs (adjusted P value 2.49E-50; Wald test) and AMNs (adjusted P value 1.21E-33). Epithelial stem cells were significantly enriched in AMN compared to normal tissue (adjusted P value 2.65E-17) or PMP (adjusted P value 1.93E-100). We determined the expression of *MUC2*, which is the gene for the predominant extracellular mucin protein in AMN and PMP. This protein is expressed in cells of the gastrointestinal tract but not found in healthy omentum (O'Connell et al., 2002a). Compared to normal appendix cells, the AMN and PMP epithelial cells showed significantly higher *MUC2* expression, with the highest levels occurring in PMP cells (ANOVA FDR P-value <2.2E-10) (Supplementary Figure S1B). We observed an increase in expression signature of gel-forming mucin genes (*MUC2*, *MUC5B*, *MUC5A*, *MUC6*, *MUC19*) in AMN and PMP samples (Nguyen et al., 2021) (Supplementary Figure S1C). Overall, our results identified a goblet cell signature from peritoneal carcinomatosis of appendiceal origin.

### Genomic instability in neoplastic goblet cells from AMNs and PMPs

**FIGURE 3:**
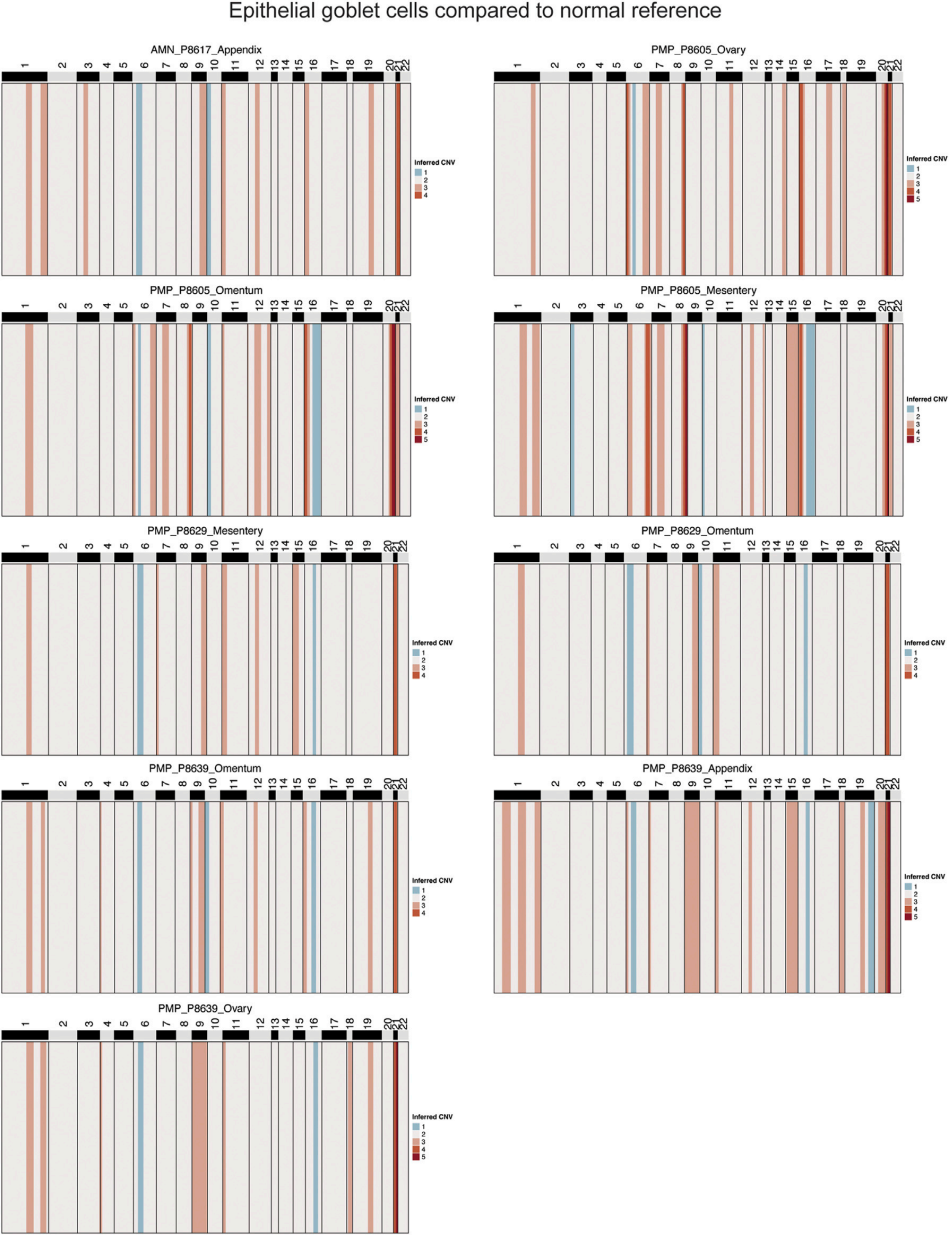
Copy number alterations in AMN and PMP. Heatmap representation of inferred single-cell CNV profiles of goblet tumor epithelial cells per respective sample compared to reference cells from normal epithelium, immune and stromal cells. CNV legend: state 1 = complete loss, state 2 = loss of one copy, state 3 = neutral, state 4 = addition of one copy, state 5 = addition of two copies, state 6 = addition of more than 2 copies.

To confirm that these goblet epithelial cells were neoplastic, we characterized the extent of somatic copy number variations (CNVs) using the inferCNV program (Patel et al., 2014; Tirosh et al., 2016) (Supplementary Material). CNVs were estimated based on average gene expression of a 10 Mb or larger windowed segment. Large CNVs with imbalances of entire chromosome arms are indicators of chromosomal instability. The CNVs present in goblet cells from AMN and PMP tumor epithelium were compared to normal appendiceal epithelial cells and other randomly sampled non-epithelial cells in the tumor microenvironment. These normal cells lacked CNVs. In contrast, the goblet epithelial tumor cells from all AMNs and PMPs had multiple CNVs (Figure 3; Supplementary Figure S2A). A gain of chromosome 9 and 21 was a frequently observed CNV across all samples including both AMNs and PMPs. We also observed gains in chromosomes 1q and 20q that have been previously identified in PMP (Alakus et al., 2014; LaFramboise et al., 2019). Altogether, these genomic instability events present in goblet epithelial cells was additional evidence that supports them being malignant.

Several genes on chromosome 21 with critical roles in tumor growth were amplified in over 88% of samples. This included genes contributing to extracellular matrix remodeling (*COL6A1*, *COL6A2*, *COL18A1*, *ADAMTS1, ITGB2*) (Winkler et al., 2020). We also identified *SOD1* and *BACH1*, involved in regulating cellular responses to oxidative stress (Papa et al., 2014; Hu et al., 2024), and transcription factor *RUNX1* that can regulate cancer cell proliferation and metastasis (Lin, 2022). Genes from chromosome 1 predicted to be amplified in 66% of samples included members of the S100 family (*S100A4*, *S100A6*, *S100A8*, *S100A9*, *S100A10*, *S100A11*, *S100A12*, *S100A13*, *S10014*, *S10016*), which are important in tumor progression and metastasis (Bresnick et al., 2015).

Chromosomal instability (CIN) is a driver of metastasis (Bakhoum et al., 2018). Using the inferred CNVs, we evaluated the fraction of genome altered per sample as a measure of CIN (Supplementary Material). PMP samples (average 15.9%) did not have a significantly greater CIN than AMN (average 13.2%) (T-test p-value 0.2798) (Supplementary Figure S2B). While this statistical analysis is underpowered given the low number of AMN samples, it raises the possibility that CIN may not be a distinguishing feature of tumor progression.

### Identification of differentially expressed genes in AMN and PMP

**FIGURE 4:**
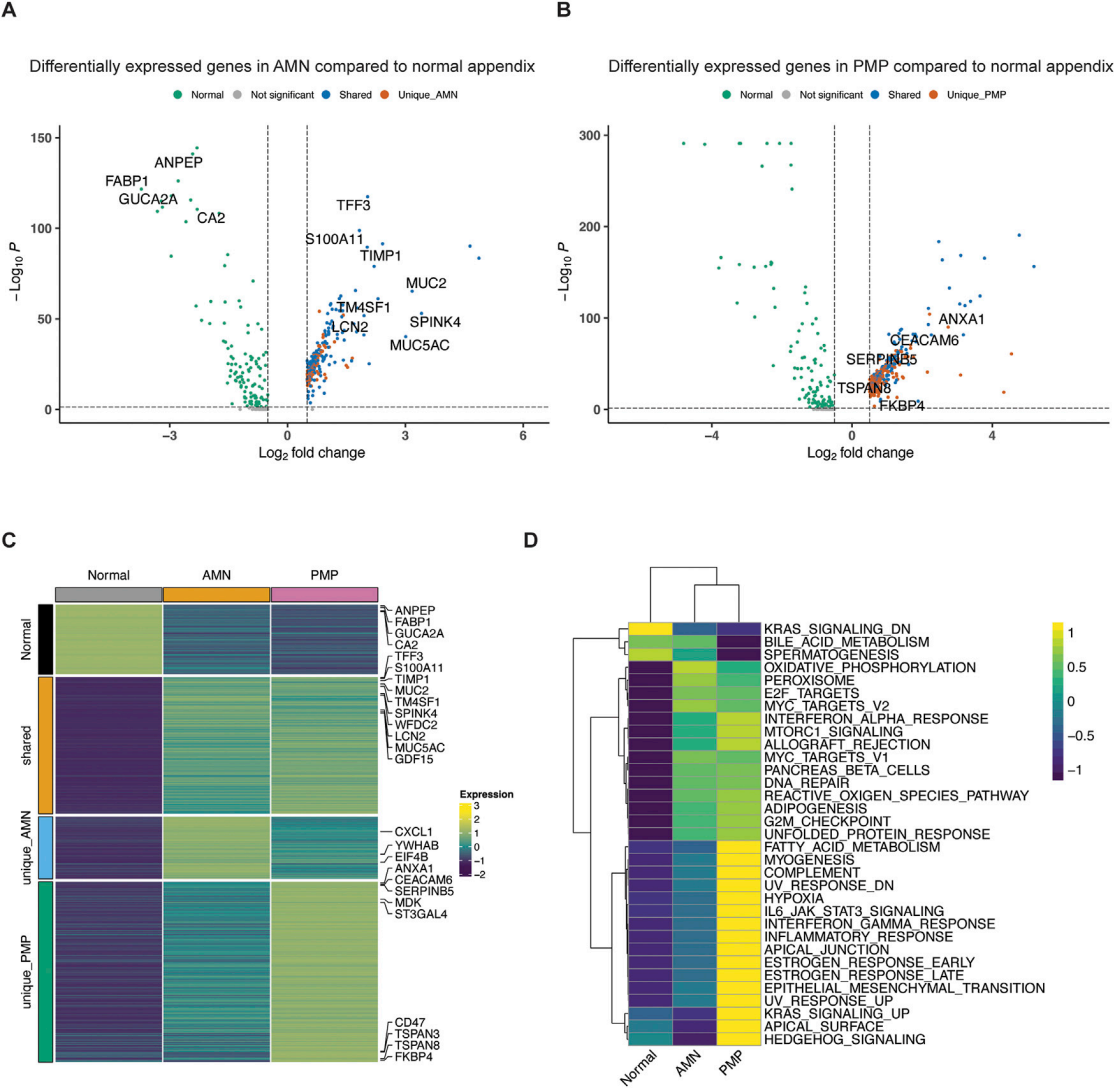
Differential gene expression in AMN and PMP. **(A, B)** Volcano plots depicting differentially expressed genes (log_2_ fold change≥0.5, adjusted *p*≤0.05) comparing **(A)** AMN to normal appendix or **(B)** PMP to normal appendix, colored by their shared and unique status. **(C)** Heatmap depicting average expression of differentially expressed genes across conditions. **(D)** Heatmap depicting average GSVA enrichment score of top Hallmark pathways per condition (ANOVA Tukey HSD p-value <0.05).

We conducted a differential gene expression analysis comparing AMN, PMP and normal appendiceal epithelial cells. Differential gene expression was based on specific criteria (Seurat Wilcoxon test, log_2_ fold change≥0.5, adjusted *p*≤0.05) when comparing epithelial cell types among the tumor and normal tissue (Barkley et al., 2022). There were four distinct gene expression signatures including: (i) a normal appendiceal signature representing genes upregulated in normal appendix compared to either AMN or PMP (86 genes); (ii) a shared signature between AMN and PMP containing genes upregulated in both compared to normal (169 genes); (iii) a unique AMN signature with genes upregulated only in AMN (77 genes); (iv) a unique PMP signature containing genes upregulated only in PMP (223 genes) (Figure 4; Supplementary Table S4).

We examined the differentially overexpressed genes and related biological pathways that were unique to each signature. The normal appendiceal expression signature included enterocyte genes (*FABP1*, *GUCA2A*, *CA2*, *ANPEP*, *AQP8*, etc.) (Figures 4A, C; Supplementary Table S4). This result is consistent with the expressed genes in normal epithelial cells in the appendix (Elmentaite et al., 2021). The AMN expression signature included the elongation factors for translation (*EIF4B*, *EEF1B2*, *EEF1D*, etc.), RNA metabolism (*YWHAB*, *LSM2*, *LSM7*, *PSMB3*) and chemokines (*CXCL1*, *CXCL2*, *CXCL3*). There was inter-sample variation in the expression of this AMN signature. It was highest in sample P8139 AMN, which also had the greatest proportion of intestinal stem cells (Figure 4C; Supplementary Figure S3A).

A set of differentially expressed genes were common to both AMN and PMP. We cite the ones that showed increased expression compared to normal appendiceal epithelium. These overlapping genes included ones related to terminal goblet cell differentiation (*TFF3*, *MUC5AC*, *MUC2*, *SPINK4*, *WFDC2* and *FCGBP*) (Figures 4A, C; Supplementary Table S4) (Elmentaite et al., 2021). Another gene set was the S100 family including *S100A11*, *S100P* and *S100A6*, which mediate tumor progression and metastasis through their roles in cell motility, invasion, and angiogenesis (Bresnick et al., 2015). The *TM4SF1* (transmembrane 4 L six family member 1) gene was overexpressed in both AMN and PMP. This gene has been implicated in cell migration and invasion. Importantly, it encodes for a cell surface protein that can potentially be targeted using monoclonal antibodies, antibody-drug conjugates and chimeric antigen receptor T cells (CAR-T) (Shen et al., 2023). Other genes associated with cancer progression and metastasis also included *TIMP1* which promotes extracellular matrix remodeling, TGF-β superfamily member *GDF15* and *LCN2*. This gene signature was observed across the majority of AMN and PMP samples (Supplementary Figure S3A).

### PMP-specific gene expression

We identified a PMP-specific gene expression signature (Supplementary Figure S3A). We cite the ones that showed increased expression. The PMP-specific genes included tetraspanin family genes (*TSPAN3*, *TSPAN8*, *TSPAN13*) and *CEACAM6* (Figures 4B, C; Supplementary Table S4). Members of the tetraspanin family have been shown to promote cancer cell motility and invasion by regulating cell signaling and adhesion (Detchokul et al., 2014). *CEACAM6* regulates cell adhesion and invasion and is a potential therapeutic target (Wu et al., 2024). Other PMP genes included members of families like the Kallikrein (KLK) family (*KLK10*, *KLK11*), the Serpin family (*SERPINB5*, *SERPINB1*, *SERPINB6*) and the sialyl-transferases *ST3GAL4 and ST6GALNAC1*. Altered sialylation by enzymes like *ST3GAL4* and *ST6GALNAC1* can contribute to tumor immune evasion and metastasis and is an emerging therapeutic strategy (Munkley, 2022).

Some genes of notable interest included *ANXA1*, *FKBP4*, *CD47* and *MDK*. These genes regulate cancer metastasis and are associated with poor prognosis in different malignancies (Gebauer et al., 2014; Shvartsur and Bonavida, 2015; Chai et al., 2016; Liu et al., 2020; Santiago-Sanchez et al., 2020; Nugteren and Samsom, 2021). However, they have not been described as playing a role in PMPs in prior studies. *ANXA1* is a phospholipid-binding protein that regulates cancer cell proliferation and metastasis (Araujo et al., 2021). *FKBP4* belongs to the immunophilin family with roles in immunoregulation, protein folding and trafficking. It is overexpressed in many tumor types with an uncharacterized role in cancer (Xiong et al., 2020). *MDK*, referred to as midkine, is a heparin-binding growth factor involved in promoting cell growth, angiogenesis, and metastasis and resistance to therapy (Filippou et al., 2020). *CD47*, often referred to as the “do not eat me” signal, is a transmembrane protein that interacts with the signal-regulatory protein alpha (SIRPα) on macrophages to inhibit phagocytosis. This molecule is an immunotherapy target (Jiang et al., 2021). Overall, these genes are candidate markers distinguishing the metastatic state of PMPs and may play a role in their cancer biology and future therapeutic target development.

### Biological pathways defining AMN and PMP epithelial cells

We observed significant differences in biology pathway activity across normal tissue, AMN and PMP (Figure 4D). We used the Gene Set Variation Analysis (GSVA) tool to evaluate pathway activation, relying on the Hallmark gene set reference (Hanzelmann et al., 2013; Liberzon et al., 2015). Compared to normal appendix cells, both AMN and PMP epithelial cells had increased pathway activity associated with E2F, MYC and G2M checkpoint functions (ANOVA with Tukey HSD p-value≤2.7e-11). These findings reflect increased proliferation in AMN and PMP tumor cells compared to normal tissue. Oncogenic mTOR signaling showed a significant increase in PMP. Both AMN and PMP had increased pathway activity for the unfolded protein response (UPR) and reactive oxygen species (ROS) generation. These results show an adaptation to managing oxidative stress which enables tumor survival and progression.

Compared to AMN, the PMP tumors had changes in metabolic pathways. This included the downregulation of oxidative phosphorylation with an increase in fatty acid metabolism. A shift towards enhanced lipid metabolism could potentially represent a survival mechanism used by metastatic cells within the microenvironment of the peritoneal niche (Nadhan et al., 2023). We also observed enrichment of the hypoxia pathway in the PMP cohort of patients, a critical factor controlling tumor cell behavior in distant metastatic niches (Rankin et al., 2016). Interestingly, these cells also had an elevated activity of inflammation pathways including increased interferon signaling, potentially reflecting responses to the metastatic microenvironment.

PMP tumors had significant upregulation of pathways associated with cancer development and progression that included epithelial mesenchymal transition (EMT), JAK-STAT and KRAS signaling. The frequency of *KRAS* mutations is very high among AMN and PMP (Alakus et al., 2014). Dysregulation in RAS, MYC and EMT pathways overlaps with previously described findings (Levine et al., 2012; Levine et al., 2016). Altogether, these pathways define the molecular and cellular mechanisms driving PMP metastasis.

### Clonal divergence and cellular signatures of PMP metastatic heterogeneity

We evaluated a subset of samples (P8605, P8629, P8639) with matched multiple metastatic sites. These organ sites included: the mesentery, fold of membrane that attaches the intestine to the wall around the stomach area; the omentum, an organ which covers the intestines; the ovaries which are a reproductive organ in females. From three patients, these matched samples had adequate tumor epithelial cell counts (20 or greater) to conduct a matched comparison analysis among the different sites. We examined the cellular heterogeneity in the tumor cells across different metastatic lesions from the same patient. First, we evaluated the similarities and differences in copy number profiles across these lesions. To understand their clonal evolution, we performed a phylogenetic analysis on these profiles. Second, we examined if this clonal divergence results in differences in gene expression properties in these lesions.

For P8605, there were three metastatic sites from the mesentery, omentum and ovary. All had gains in chromosomes 8, 12, 16 and 20 (Supplementary Figure S4A). Only the mesenteric site had a gain in chromosomes 1 and 15. Based on these chromosomal alterations, we generated a phylogenetic tree to map the evolution of tumor subclones (Supplementary Figure S4B). The root branch represented the normal reference diploid cells while the other branches represented each metastatic site. The mesenteric site diverged from the ovarian and omental metastasis and had the largest distance from the normal reference. This distance reflected the additional aneuploidy events in the mesenteric metastatic lesion. Each site also had distinct gene expression properties. For the mesenteric metastasis, we observed significantly increased expression of metastasis-related genes *ANXA1* and *KLK10* compared to the omental and ovarian implants (adjusted p-value <0.05) (Supplementary Figure S4C). The omental metastasis had higher expression of the goblet cell differentiation markers *FCGBP* and *TFF3*. The three sites differed in their expression of *GNAS*, a G protein-coupled receptor-regulated adenylyl cyclase. Among PMPs, this gene frequently has mutations and regulates mucin production (Lin et al., 2020). The P8605 tumor underwent targeted cancer gene sequencing and had a mutation in *GNAS* (Supplementary Table S1). Importantly, our analysis discovered a variation in *GNAS* expression level across multi-lesion metastasis in the same patient.

For P8629, there were two metastatic sites from the mesentery and omentum. Only the mesenteric metastasis had amplifications in chromosomes 1 and 16 (Supplementary Figure S5A). This increased aneuploidy was also observed in the phylogenetic analysis. The mesenteric lesion had a greater distance from normal reference compared to the omental lesion (Supplementary Figure S5B). The omental metastasis had a higher expression (adjusted p-value <0.05) of goblet cell differentiation markers *TFF3*, *MUC2* and *SPINK4* (Supplementary Figure S5C). Conversely, the mesenteric lesion had enriched expression of the keratin family genes (*KRT7*, *KRT17*) involved in cancer progression as well as metastasis signature associated gene *CEACAM6*.

For P8639, there were two metastatic sites from the omentum and the ovary, as well as the primary appendiceal cancer. All lesions had an amplification in chromosome 21 (Supplementary Figure S6A). Only the appendiceal lesion had amplifications in chromosomes 1 and 19. In the phylogenetic analysis, the ovarian and omental lesions were more closely related given the similarities in their CNV profiles (Supplementary Figure S6B). The appendiceal lesion diverged from these owing to differences in CNV alterations. Compared to the primary appendix site, the metastatic tumors had differential expression of the following genes: *TCN1* which encodes the transcobalamin 1 protein that binds the B12 vitamin and is associated with poor prognosis and metastasis in colon cancer (Liu et al., 2020); the goblet cell marker *WFDC2*; *GNAS* which is a driver of appendiceal cancer (Supplementary Figure S6C).

Hence, among PMP tumors in different anatomic locations from the same patient, we identified metastatic clonal divergence. This result confirmed that clonal heterogeneity was present among the different tumor sites of the same patient. Moreover, this heterogeneity was also present at the gene expression level. PMP metastatic sites from the same patient had different properties of stemness, goblet cell identity and metastasis differentiation.

### Protein markers expressed among PMP tumors

As we described across the different tumors, the PMP epithelial cells have a specific gene expression signature indicating goblet cell differentiation as well as a metastatic gene expression signature. To validate the expression of the genes as proteins, we used a combination of immunohistochemistry (IHC) and mass spectrometry on a subset of the PMP tumors and ascites respectively. For the IHC study, we used antibodies staining for the MUC2 and AGR2 protein which had overexpression in both AMN and PMP tumors. Also, we included an antibody staining for the ANXA1 protein–this gene was found to be overexpressed among the PMP metastases.

**FIGURE 5:**
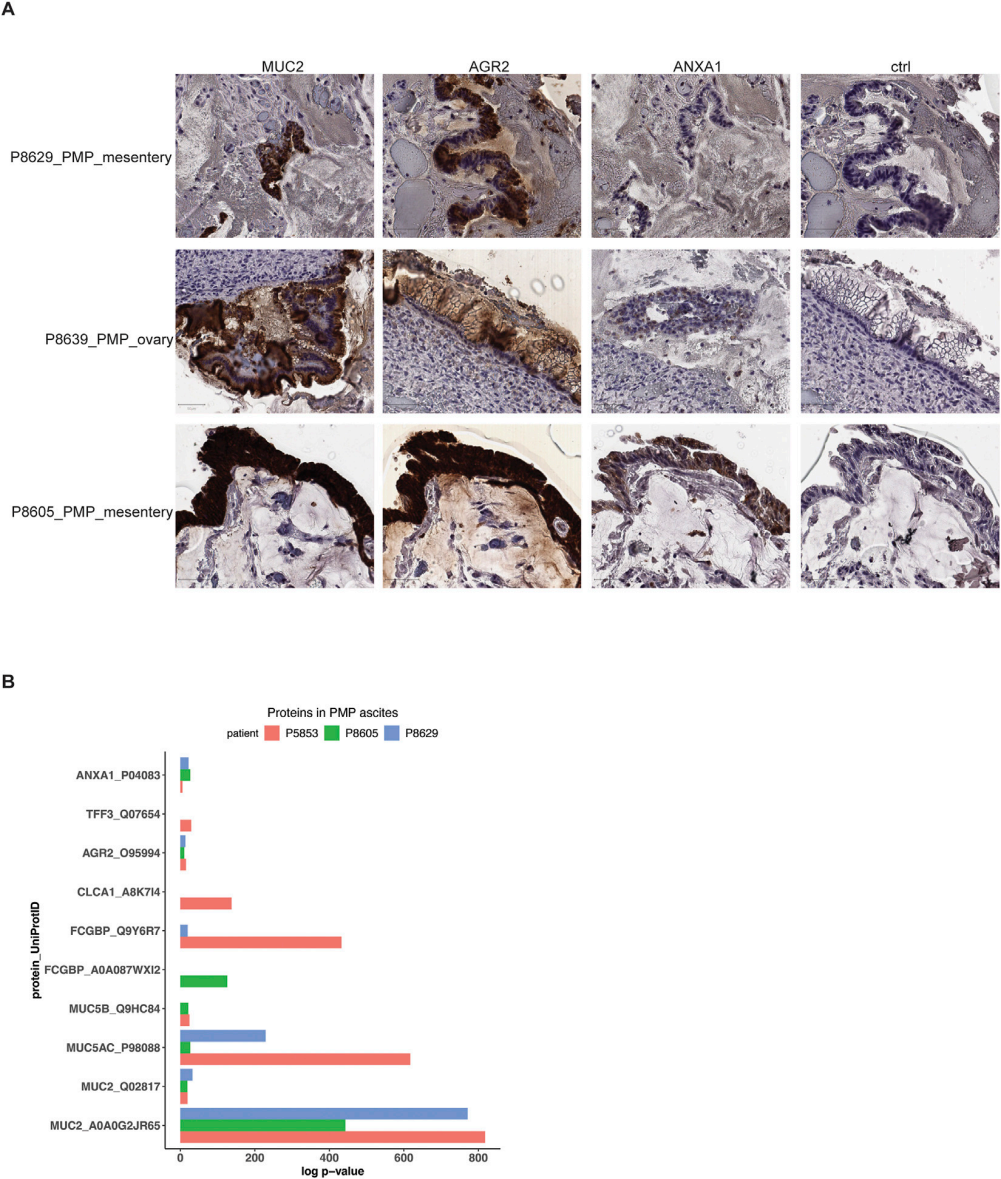
Protein expression in PMP lesions and ascites. **(A)** Representative images of immunohistochemical staining of respective tumor samples for MUC2, AGR2, ANXA1 or representative isotype control. Scale bar = 50 μm. **(B)** Selected proteins detected in mass spectrometry of PMP ascites samples with their detection p-value.

For the three PMP tumors (P8629, P8639 and P8605), MUC2 and AGR2 had intense staining among the epithelial tumor cells (Figure 5A). All tumors expressed ANXA1 but in varying degrees, and with less intensity than MUC2 or AGR2. The tissues tested included mesentery and ovary, which do not contain goblet cells, as these are cells from the gastrointestinal mucosal lining stained by MUC2 and AGR2. Tissue from organs that are not part of the digestive system do not express these proteins. Cells with these proteins represent tumors that have metastasized. These results show that all markers were expressed among the PMP tumor epithelial cells.

Three patients with PMP (P5853, P8605 and P8629) had malignant ascites–this abnormal fluid buildup occurs as a direct result of tumor cell accumulation. Specifically, goblet cells are secretory cells of the internal lumen of the gastrointestinal tract and release proteins and other products which contributes to ascites. Using mass spectrometry, we determined if protein products of goblet cells, normally found inside the gastrointestinal lumen following their secretion, were secreted into the ascites by the tumor cells with a goblet cell signature. We identified the likelihood that peptide spectrum matches arise by random chance based on a probabilistic model (Bern et al., 2012) (Supplementary Material). Next, we used a confidence threshold requiring a detected protein to have a log p-value at least 2.0-fold lower than the log p-value of the top decoy protein (Supplementary Material) (Elias and Gygi, 2007). Based on the mass spectrometry results, the P5853 sample had 299 proteins, the P8605 sample had 300 proteins and the P8629 sample had 276 proteins (Supplementary Table S5).

Across all three patient samples, we observed significant expression of mucin proteins MUC2, MUC5AC and MUC5B, goblet cell markers AGR2 and FCGBP and metastasis marker ANXA1 (Figure 5B; Supplementary Table S5). In addition, P5853s ascites had goblet cell secretory products TFF3 and CLCA1 (Kim and Ho, 2010). Overall, these results supported a goblet cell identity for the PMP tumors. These results demonstrated a direct role for these cells in the secretion and production of the gelatinous malignant ascites.

### Validating the gene expression signature of PMP tumor cells

**FIGURE 6:**
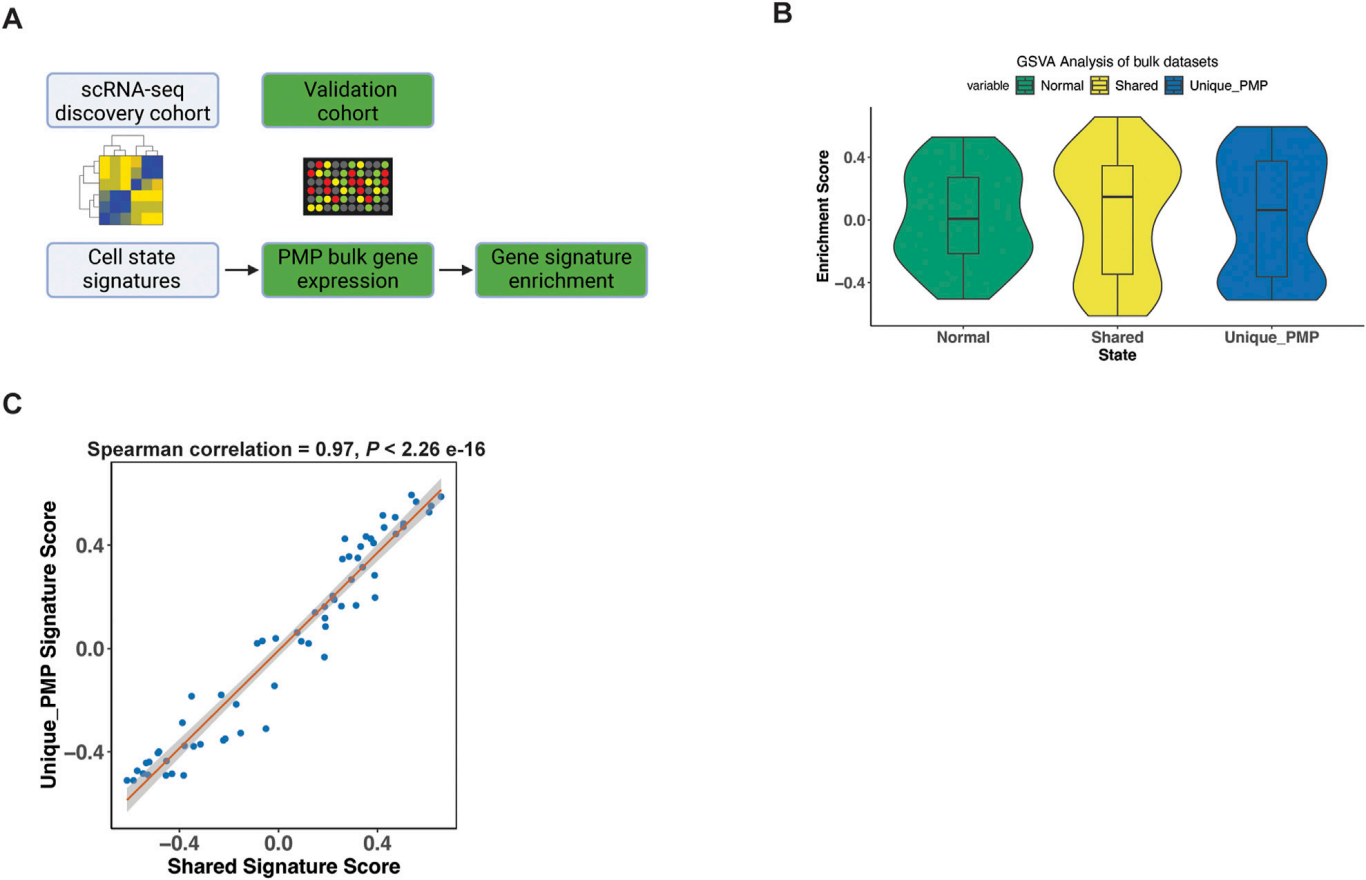
Gene signature validation in external datasets. **(A)** Schematic representation of signature enrichment analysis. **(B)** GSVA scores for respective tumor epithelial cell state in validation bulk dataset samples. **(C)** Scatter plots showing Spearman correlation of respective tumor epithelial cell state signatures across all samples from the validation bulk datasets.

We validated our scRNA-seq results using microarray gene expression from 63 PMP tumors (Levine et al., 2016). For this analysis, we used the gene expression signatures representing normal, goblet cell differentiation shared between AMN and PMP and metastasis signature unique to PMP identified in our previous analysis (Figure 6A). We applied a GSVA analysis which provided a quantitative enrichment score.

The PMP tumors had positive GSVA enrichment scores for the normal (50.8%), shared goblet cell signature (55.6%), and the PMP metastatic state (58.7%) (Figure 6B). Hence, the expression signatures from our single-cell RNA-seq analysis were detected among other PMPs. Conventional RNA-seq does not discriminate gene expression from single cells such as tumor epithelium, thus these percentages reflect decreased sensitivity for detecting fold changes in specific genes. The goblet state enrichment scores were significantly correlated with metastasis gene signature scores (Figure 6C) across all samples (Spearman co-efficient 0.97, p < 2.2e-16). This indicated that tumors co-expressed both goblet cell and metastasis gene signatures, validating our scRNA-seq findings.

### Infiltrating lymphocytes in the AMN and PMP cellular microenvironment

**FIGURE 7:**
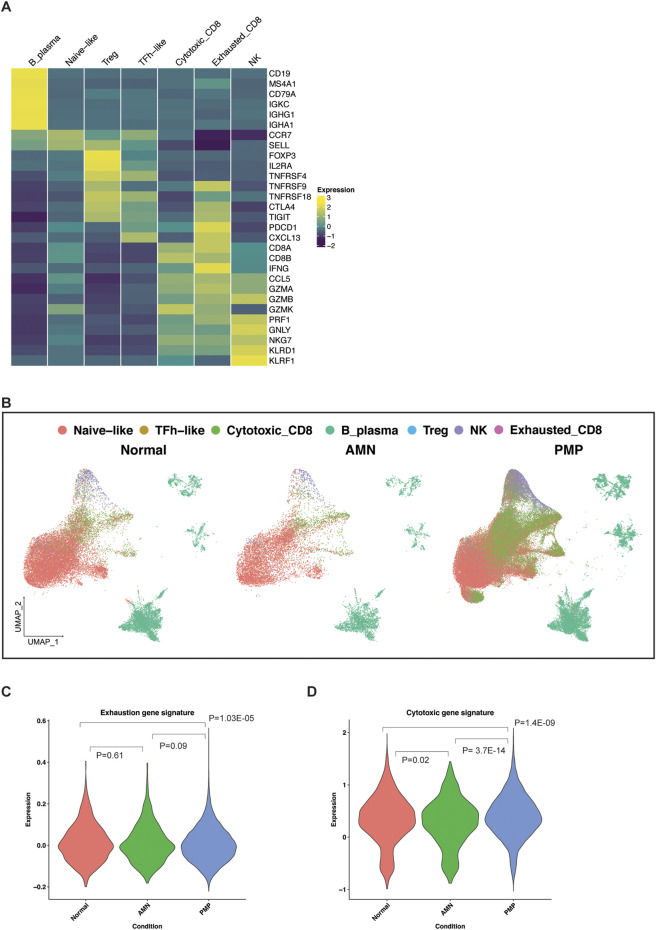
Lymphocytes in the TME of AMN and PMP samples. **(A)** Heatmap depicting average expression levels of respective genes in lymphocyte subsets from normal, AMN and PMP samples. **(B)** UMAP representation of dimensionally reduced data following batch correction and graph-based clustering of all datasets annotated by lymphocyte subsets across normal, AMN and PMP samples. **(C, D)** Violin plots depicting expression of **(C)** exhaustion or **(D)** cytotoxicity gene signature with ANOVA Tukey HSD p-value in normal, AMN and PMP infiltrating CD8 T cells.

We characterized the infiltrating lymphocytes in the AMN and PMP tumor microenvironment (Figure 7). To determine the different immune cell types, we used a gene expression reference of infiltrating immune cells into tumors–these gene lists include established expression markers for specific cell types (Supplementary Material) (Nieto et al., 2021). Across all samples, we identified B and plasma cells (*MS4A1*, *CD19*, etc.) (Figures 7A, B). We detected T cell subsets including naïve-like (*CCR7*, *SELL*), regulatory T (Tregs) (*FOXP3*, *IL2RA*, etc.) and follicular helper-like cells (TFh-like) (*CXCL13*, *TNFRSF18*, *CTLA4, etc.*). We identified cytotoxic CD8 T (*CD8A*, *CD8B*, *CCL5*, etc.), activated-exhausted CD8 T cells (*CD8A*, *CD8B*, *CXCL13*, *PDCD1*, *CTLA4*, etc.) and NK cells (*KLRD1*, *KLRF1*, *GNLY*, etc.) (van der Leun et al., 2020; Nieto et al., 2021). A recent study in PMP using spatial gene expression analysis supports the presence of tumor infiltrating lymphocytes (Su et al., 2020). While the absolute number of cells varied by sample (Supplementary Table S6), all tumors contained TFh-like CD4 T, cytotoxic CD8 T and NK cells. Interestingly, very few cells mapped to the exhausted CD8 phenotype.

The appendix and omentum are lymphocyte rich organs. Infiltrating CD8 T cells in AMN and PMP tumor microenvironment may be anti-tumor cells or bystander cells such as those occur in normal tissue. Infiltrating lymphocytes in AMN and PMP largely co-clustered with those in normal tissue (Figure 7B), indicative of similar gene expression properties. An increase in exhaustion or dysfunction has been linked to CD8 T cells that recognize tumor antigens (van der Leun et al., 2020; Meier et al., 2022). We evaluated the gene expression signature of exhausted T cells (Zheng et al., 2017) (Supplementary Material, Supplementary Table S7). Compared to normal tissues, AMN tumors had no significant differences in the number of CD8 T cells with an exhaustion phenotype (Figure 7C). However, PMP tumors did show a significant reduction of exhausted CD8 T cells.

Next, we assessed the expression of a gene signature of cytotoxic effector genes in these CD8 T cells (Tirosh et al., 2016; Guo et al., 2018) (Supplementary Material, Supplementary Table S7). In the AMN tumor microenvironment, CD8 T cells had a reduction in cytotoxic gene expression compared to normal tissue (Figure 7D). In the PMP tumor microenvironment, T cells had increased cytotoxic gene expression. These PMP-based infiltrating CD8 T cells in PMP continued to have high cytotoxic potential. These cells do not express markers of anti-tumor activity. However, their cytotoxic potential could be harnessed further for strategies that aim to convert bystander cells into infiltrating cells that can contribute to anti-cancer activity (Meier et al., 2022). This is further supported by recent studies that demonstrated an *ex vivo* T cell activation in PMP tumors (Flatmark et al., 2021; Forsythe et al., 2021; Weitz et al., 2022).

## Discussion

It has been a challenge to define the cellular characteristics of AMN and PMPs. AMN progression to PMP and the underlying cellular changes accompanying it are not fully characterized. These tumors pose challenges for translational studies that include rarity of these tumors, their low cellularity and complex gelatinous composition. The epithelial cell population is often diluted among the other cell types and mucinous components of these tumors. To overcome these challenges, we used scRNA-seq on a set of AMNs without peritoneal dissemination and PMPs with peritoneal dissemination. We identified the cellular features of the tumor epithelial cells present in gelatinous appendiceal tumors with and without peritoneal dissemination.

An important aspect of our study was determining the origin and the distinct populations of AMN and PMP neoplastic cells in the tumors evaluated. The discovery of MUC2 expression implicated an enteric rather than ovarian origin for PMP tumors (O'Connell et al., 2002a). However, prior studies have not firmly established whether this was due to acquisition of MUC2 expression by non-gastrointestinal tumor cells and/or cells with an intestinal lineage origin. One study found that appendiceal tumors of diverse histology had enrichment of goblet-like tumor cells in the epithelial lineage (Bui et al., 2023). We leveraged single cell genomics to define the features of the epithelial tumor cells in AMN and PMP tumors. Our study focused on tumors with mucinous pathology and conclusively established that the epithelial tumor cells have a goblet cell identity. We observed tumor cells with goblet cell features in peritoneal metastases of tissues that lack intestinal epithelial cells (e.g., omentum, small bowel mesentery, ovary).

Prior studies have reported detecting MUC2 and MUC5AC protein in PMP tissue and ascites (O'Connell et al., 2002b; Mall et al., 2007). Here, we identified multiple goblet cell-specific secretory products in the ascites of PMP patients (e.g., AGR2, MUC2, FCGBP). More importantly, expression of these proteins corresponded to increased gene expression in the tumor epithelial cells of patients. These tumor cells were present in PMP implants in non-gastrointestinal organs such as the peritoneum and ovary. In addition, our review of other translational studies analyzing the protein composition of ovarian and gastric malignant ascites were different than the protein composition of AMN or PMP-derived ascites (Shender et al., 2014; Jin et al., 2018). Altogether, these results for the first time show that tumor cells with a goblet cell signature in appendiceal mucinous tumors contribute to the mucinous phenotype of this cancer.

We identified distinct gene expression signatures for the different population of neoplastic cells found in AMN and PMP tumors. This included common gene expression features between AMN and PMP tumors indicative of goblet cell differentiation and tumor progression, and a metastasis associated gene signature unique to PMP tumors. We validated the PMP gene expression signature using an independent data set of PMP tumors (Levine et al., 2016). Importantly, we identified new genes associated with this metastatic cell state including *ANXA1*, Kallikrein family, *FKBP4,* Serpin family, *CD47*, *MDK* and the sialyl-transferases *ST3GAL4* and *ST6GALNAC1*. These genes have been implicated in cancer development and metastasis among other types of primary malignancies but not in appendiceal mucinous neoplasms. Further studies and characterization of these genes may provide greater insight into the biology of PMP metastasis and their prognostic significance in disease progression.

Current gold standard therapy for PMP patients consists of cytoreductive surgery and HIPEC (Bartlett et al., 2019). Meanwhile, systemic chemotherapy has a more limited role and is considered in high grade tumors or in a palliative role. This study identified activated signaling pathways for potential target therapy including: tetraspanin and kallikrein family genes, MYC, PI3K-AKT-mTOR and RAS signaling pathways, aberrant glycosylation, altered lipid metabolism, and targetable cell surface proteins such as TM4SF1 and CD47. Interestingly, a recent study found differences in metabolic pathways, in particular lipid, glutathione and oxidative phosphorylation, between appendiceal tumor of LAMN and adenocarcinoma histology (Hanse et al., 2023). Other studies have shown that AMN and PMP tumors have low frequency mutations in *SMAD4*, *ATM*, *PIK3CA*, *AKT* and *JAK3*. We also identified heterogeneity in gene signature expression within the same tumor and at different metastatic sites in the same PMP patient. This heterogeneity maybe a source of resistance to treatment regimens. The identification of additional targets and signaling pathways in this study may allow tailoring or multi-agent therapy development for patients in the future.

Both AMN and PMP’s immune TME contained infiltrating T cells. Our results indicated that these cells are like those in normal tissue. Additional studies using T-cell receptor sequencing could further clarify if these cells are bystander cells or have tumor-specificity. Importantly, these cells retained cytotoxic gene expression suggesting they could be targeted for therapeutic purposes. Recent studies have demonstrated that PMP tumors can respond *ex vivo* to immune checkpoint blockade or neoantigen vaccination (Flatmark et al., 2021; Forsythe et al., 2021; Weitz et al., 2022). Interestingly, a recent study looking at the TME of patients pre and post HIPEC noticed improved progression free survival in patient with higher CD8+/PD-L1+ when compared to lower values (Su et al., 2024). Further functional characterization of this lymphocyte infiltrate will help clarify the role of these cells in anti-cancer immunity. Strategies that improve cancer cell killing by bystander cells including TCR-independent innate-like killing could also be explored further for these patients (Meier et al., 2022).

## Data Availability

The datasets presented in this study can be found in online repositories. The names of the repository/repositories and accession number(s) can be found below: https://www.ncbi.nlm.nih.gov/gap/, phs001818. Cell-gene matrices are available at https://dna-discovery.stanford.edu/research/datasets/.
